# Spelling Changes and Fluorescent Tagging With Prime Editing Vectors for Plants

**DOI:** 10.3389/fgeed.2021.617553

**Published:** 2021-03-04

**Authors:** Li Wang, Hilal Betul Kaya, Ning Zhang, Rhitu Rai, Matthew R. Willmann, Sara C. D. Carpenter, Andrew C. Read, Federico Martin, Zhangjun Fei, Jan E. Leach, Gregory B. Martin, Adam J. Bogdanove

**Affiliations:** ^1^Plant Pathology and Plant-Microbe Biology Section, School of Integrative Plant Science, Cornell University, Ithaca, NY, United States; ^2^Department of Bioengineering, Faculty of Engineering, Manisa Celal Bayar University, Manisa, Turkey; ^3^Boyce Thompson Institute for Plant Research, Ithaca, NY, United States; ^4^Plant Pathogen Interaction, National Institute for Plant Biotechnology (ICAR), New Delhi, India; ^5^Plant Transformation Facility, School of Integrative Plant Science, Cornell University, Ithaca, NY, United States; ^6^Department of Agricultural Biology, Colorado State University, Fort Collins, CO, United States

**Keywords:** prime editing, plant genome editing, fluorescent tagging, split GFP, *Oryza sativa*, Arabidopsis, *Nicotiana benthamiana*

## Abstract

Prime editing is an adaptation of the CRISPR-Cas system that uses a Cas9(H840A)-reverse transcriptase fusion and a guide RNA amended with template and primer binding site sequences to achieve RNA-templated conversion of the target DNA, allowing specified substitutions, insertions, and deletions. In the first report of prime editing in plants, a variety of edits in rice and wheat were described, including insertions up to 15 bp. Several studies in rice quickly followed, but none reported a larger insertion. Here, we report easy-to-use vectors for prime editing in dicots as well as monocots, their validation in *Nicotiana benthamiana*, rice, and Arabidopsis, and an insertion of 66 bp that enabled split-GFP fluorescent tagging.

## Introduction

Prime editing (PE) is an adaptation of the CRISPR-Cas system that uses a Cas9(H840A)-reverse transcriptase (RT) fusion and a guide RNA (pegRNA) amended with template and primer binding site (PBS) sequences to achieve RNA-templated conversion of the target DNA, allowing specified substitutions, insertions, and deletions (Anzalone et al., 2019). A second version of the system, PE2, incorporates an improved, engineered RT, and a third, PE3, adds to that a sgRNA directing a nick to the non-edited strand to drive its conversion (Anzalone et al., 2019).

Prime editing in plants was first reported by Lin et al. (2020), who achieved a variety of edits in rice and wheat. Several other studies in rice and one each in tomato, potato, and maize have been published since (Butt et al., 2020; Hua et al., 2020; Jiang et al., 2020; Li et al., 2020; Lu et al., 2020; Tang et al., 2020; Veillet et al., 2020; Xu et al., 2020a,b). While the editing efficiencies ranged from 1.55 to 31.3% in rice (Butt et al., 2020; Hua et al., 2020; Li et al., 2020; Lin et al., 2020; Tang et al., 2020; Xu et al., 2020a,b), the highest efficiency observed in tomato was 1.66% (Lu et al., 2020). Potato was similar to tomato (Veillet et al., 2020). The highest efficiency overall, 53.2%, was in maize, obtained by optimization of pegRNA expression (Jiang et al., 2020). In contrast to results in mammalian cells (Anzalone et al., 2019), PE3 did not increase editing efficiency in plants relative to PE2 (Butt et al., 2020; Lin et al., 2020; Veillet et al., 2020; Xu et al., 2020a). In some studies, in fact, PE2 yielded a much higher editing efficiency than PE3 (Jiang et al., 2020; Tang et al., 2020). Unintended target site mutations including insertions, deletions, and substitutions were reported in almost all of the plant PE studies, though no unintended insertions or deletions were reported in maize (Jiang et al., 2020).

The largest targeted insertion by PE reported to date was in human cells, a 44-bp *loxP* tag (Anzalone et al., 2019). In plants, the largest insertion reported was 15 bp; attempts at larger insertions, up to 60 bp, were not successful (Lin et al., 2020). The apparent constraint on insertion length using prime editing potentially limits its application for introducing translational fusions, for example to a fluorescent protein for localization.

Here, we report easy-to-use vectors for PE in dicots and monocots, their validation in three plant species, and an insertion of 66 bp that enabled split-GFP fluorescent tagging. The vectors are suitable for PE2 or for PE3.

## Method

### Vector Construction

The binary vector for PE in dicots, pPPED, was constructed by replacing the 35S promoter and *Cas9* in binary vector p201N (Jacobs et al., 2015) with a double 35S promoter and *Cas9(H840A)* from pMOD_A0301 (Cermak et al., 2017) plus a commercially synthesized (Integrated DNA Technologies, Coralville, IA USA), tomato codon-optimized 34-aa flexible linker and engineered RT sequence (Anzalone et al., 2019), then adding a Gateway destination cassette (Thermo-Fisher, Waltham, MA USA). The smaller, non-binary vector for transfection or bombardment, pPPEDs, was created by moving these components into pBluescript KS(-). The binary vector for PE in monocots, pPPEM, was created by mutating pUbi-Cas9, which already contains a Gateway destination cassette (Zhou et al., 2014), to encode Cas9(H840A) using the Q5 Site-Directed Mutagenesis Kit (New England Biolabs, Ipswich, MA USA), then adding synthesized linker and RT sequence, optimized for rice. The entry vector for RNA modules, pPEG, was created by inserting into pCR8/GW/TOPO (Thermo-Fisher) a CmYLCV promoter-driven cassette containing two *Bsa*I sites across a short spacer for introducing module elements by Golden Gate cloning (Engler et al., 2008), with a gRNA scaffold downstream, together flanked one each side by an Arabidopsis pre-tRNA(Gly) gene sequence, and followed by the Arabidopsis *HSP18.2* gene terminator (sequences from Stavolone et al., 2003; Nagaya et al., 2010; Xie et al., 2015; Cermak et al., 2017) and, further downstream, a unique *Bae*I site downstream for inserting additional elements. Final PE constructs were prepared by introducing synthesized DNA sequence for the pegRNA with scaffold followed by tRNA(Gly) and an sgRNA spacer, and with a *Bsa*I site and compatible sequence at each end, into pPEG by Golden Gate reaction, then transferring the resulting module into pPPED, pPPEDs, or pPPEM by LR recombination.

### *Nicotiana benthamiana* Agroinfiltration Assay

Transformants of *Agrobacterium tumefaciens* strain GV3101 carrying the helper plasmid pMP90 and pPPED or derivatives were grown in yeast extract peptone medium with the appropriate antibiotics overnight at 30°C. Bacteria were resuspended in infiltration buffer (10 mM MgCl_2_, 10 mM MES [pH 5.6], and 200 mM acetosyringone) and were incubated with shaking for 2–4 h in the dark at room temperature. Bacterial cultures were then centrifuged, washed, resuspended in infiltration buffer, and adjusted to the final OD_600_ indicated in each experiment. Leaves of 5-week-old *Nicotiana benthamiana* plants were infiltrated using a needle-less syringe and were placed in a growth chamber (24°C day and 22°C night). Cell death was scored and photographed 6 or 12 days after infiltration. For amplicon sequencing, tissue was collected 6 days after infiltration, and DNA was extracted using the DNeasy Plant Mini Kit (Qiagen, Hilden, Germany), then PCR was performed using 50 ng of DNA and specific primers (Supplementary Table 1) with KAPA HiFi HotStart ReadyMix (Roche, Basel, Switzerland) in 25 μL reactions using the recommended protocol.

### Rice Protoplast Assay

Seventy Oryza sativa ssp. japonica cv. Nipponbare seeds were sanitized in 70% ethanol for 2 min, followed by 40% commercial bleach for 30 min, then rinsed 5 times in autoclaved distilled water and dried on sterile filter paper. The sterile rice seeds were planted in 10 cm diameter glass jars on half MS media incubated in a growth chamber under a cycle of 12 h light at 28°C and 12 h dark at 25°C. After 12 days, the seedlings were used to isolate protoplasts as described (Shan et al., 2014) with the following modifications: filter-sterilized enzyme solution was added to the strips immediately (pre-incubation in 0.6 M mannitol was omitted), the strips were incubated in the dark for 7–8 h with gentle shaking at 100 rpm, and, after enzymatic digestion, W5 solution [2 mM MES (pH5.7), 154 mM NaCl, 125 mM CaCl_2_, 5 mM KCl] was added and the digest shaken gently for 1 min to release the protoplasts; additionally, all centrifugation was carried out at 150 x g and supernatants were decanted by pouring. Protoplasts were quantified using a hemocytometer, and transfection was carried out using PEG as described (Shan et al., 2014) with the following modifications: the number of protoplasts used per transfection was 10^6^, and in the final step, protoplasts were resuspended in 2 ml MMG solution [4 mM MES (pH5.7), 0.4 M mannitol, 15 mM MgCl_2_] (instead of WI medium) before being incubated in a 6-well plate at 25°C in the dark for 2 days. Plasmid DNA for transfection was prepared using the HiSpeed Plasmid Maxi Kit (Qiagen) according to manufacturer instructions. For transfections with pPPEM or a derivative only, 15 μg was used. For transfections with an added pPEG construct, 15 μg of the pPPEM derivative and 4 μg of the pPEG construct were used. To estimate transformation efficiency, separately, protoplasts were transfected with 4 μg of pMOD_C3001 (Cermak et al., 2017) and 11 μg of pPEG (as carrier DNA) and imaged under an upright BX-50 fluorescence microscope (Olympus Corporation, Tokyo, Japan). For amplicon digests and sequencing, genomic DNA was isolated using the CTAB method (Allen et al., 2006), then PCR was performed using 40 ng of DNA and specific primers (Supplementary Table 1) with Q5 high-fidelity DNA polymerase (New England Biolabs) in 25-μL reactions using the recommended protocol. Selected PCR products were digested using *Bst*Z17I (New England Biolabs) and analyzed by 1% agarose gel electrophoresis.

### Arabidopsis Protoplast Assay

Arabidopsis protoplast transient expression experiments were done according to a published protocol (Yoo et al., 2007) except for a few modifications that follow. Plants were grown in Lambert Mix 1 (LM-1) in a Percival growth chamber at 22°C under a cycle of 16 h light and 8 h dark. Mesophyll protoplasts were isolated from fully-expanded leaves 5–8 of 4-week-old non-flowering plants. Digestion of 0.5–1.0 mm leaf strips was performed for 2 h in 1.5% cellulase R10 and 0.4% macerozyme R10 (Yakult Pharmaceutical Industry Company, Tokyo, Japan), 0.4 M mannitol, 20 mM KCl, 20 mM MES, pH 5.7, 10 mM CaCl_2_, 0.1% BSA. The digest was then diluted 1:1 with W5 solution and filtered through Miracloth to remove undigested cellular debris. Following washing steps, the protoplasts were quantified using a hemocytometer and resuspended in MMG solution to 200,000 cells per ml. For each transfection, ~50,000 protoplasts and 50 μg of plasmid DNA, prepared using the HiSpeed Plasmid Maxi Kit (Qiagen) according to manufacturer instructions, were used. Following transfection, the cells were transferred to WI solution as described (Yoo et al., 2007) except that following centrifugation, ~100 ul of the buffer was left in the tube and used to resuspend and transfer the cells to one well of a 12-well culture dish having one 1 ml of WI solution. The cells were incubated at room temperature for 24 h prior to microscopy or centrifugation for DNA extraction. Transfection efficiency was estimated and DNA extraction was carried out as described for the rice protopasts, above, except that a 35S:eGFP construct was used (Chiu et al., 1996) and PCR was carried out with 10 ng of template DNA.

### Amplicon Sequencing

Amplicons for sequencing (each <500 bp) were purified after 1% agarose gel electrophoresis by using the Monarch Gel Extraction Kit (New England Biolabs) and quantified with a NanoDrop 2000 spectrophotometer (Thermo Fisher). 500 ng of each was sent for commercial sequencing (Genewiz, South Plainfield, NJ USA) by indexed Illumina MiSeq paired-end (2 × 250 bp) reads. Reads were analyzed using CRISPResso2 v.2.0.37 (Clement et al., 2019). To determine the number of reads reflecting perfectly edited target DNA (“perfect-edit reads,” with the edit and no other change) CRISPResso2 was run in HDR mode using a quantification window spanning 2 bp to the outside of the pegRNA and sgRNA nick sites and everything in between, and the number of perfect-edit reads was taken from the resulting alleles table (rather than being taken as the number of HDR reads, which does not exclude reads with a substitution or indel within the edit). Editing efficiency was calculated as (perfect-edit reads/total mapped reads)^*^100 divided by transfection efficiency and averaged across replicates (Supplementary Table 2). To calculate the proportion of “edit variants,” reads containing the intended edit but also at least one other difference from the original sequence within the large quantification window, the total number of edit reads was first determined by a separate CRISPResso2 analysis using a smaller window that examined only the intended edit, then the number of perfect-edit reads was subtracted from that total and the result divided by the number of perfect-edit reads.

## Results and Discussion

### Vectors and Strategy for PE in Plants

**Figure 1:**
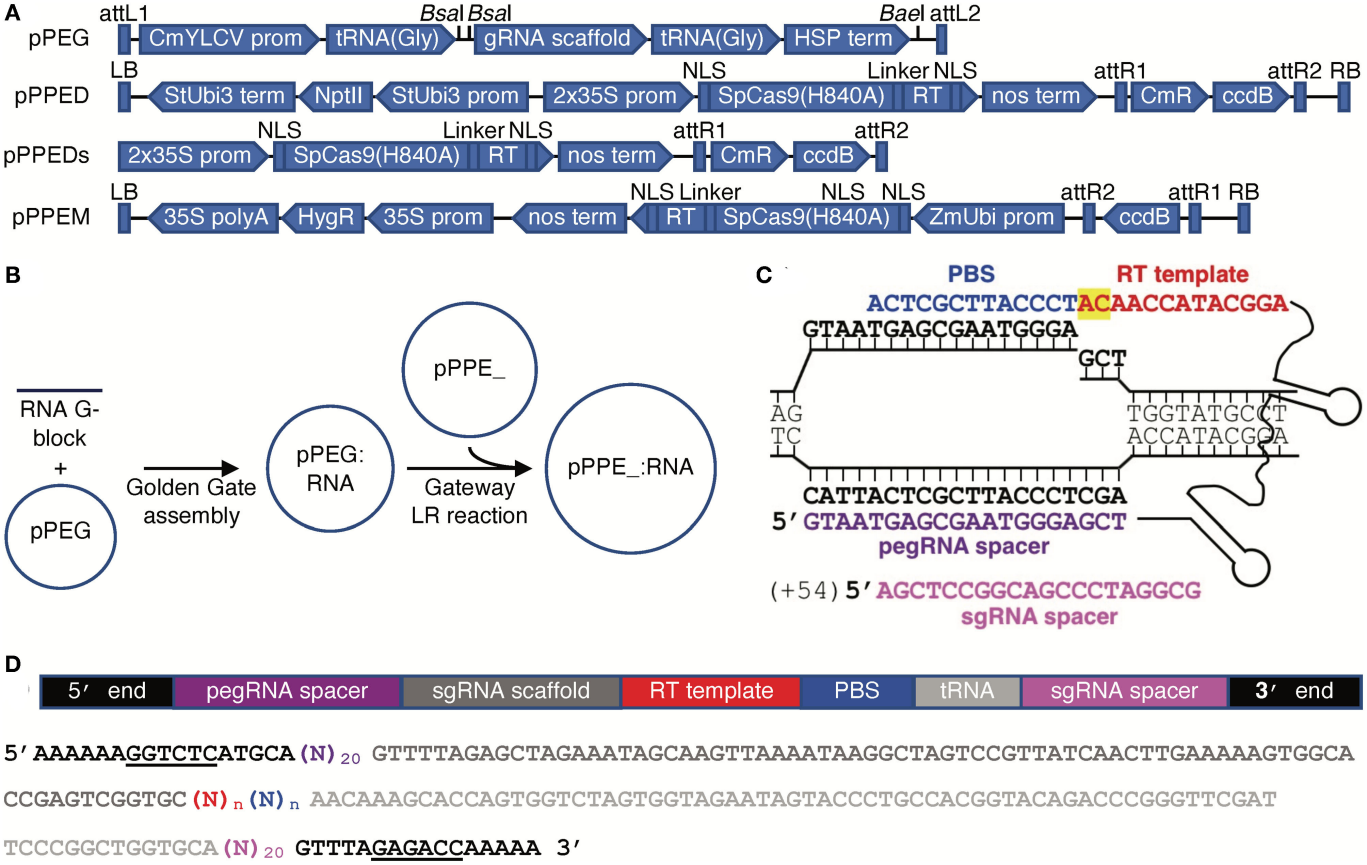
Vectors and construct assembly for PE in plants. **(A)** Features of each vector (vectors and annotated sequences available at www.addgene.org). **(B)** Workflow for generating PE constructs. **(C)** A pegRNA on its target, showing the PBS (blue), RT template (red, with the edit highlighted in yellow), spacer (purple), and nick site, and, for PE3, the sgRNA (magenta) with the position of its nick site relative to the pegRNA-mediated nick in parentheses. **(D)** Schematic and color-matched sequence of an RNA module including compatible ends for Golden Gate cloning into pPEG (*Bsa*I sites underlined), pegRNA spacer, sgRNA scaffold, RT template and PBS (of unspecified lengths, *n*), and optionally a tRNA(Gly) and sgRNA spacer.

We created a binary vector for use in dicots, pPPED, and a binary vector for use in monocots, pPPEM; we also created a smaller, non-binary version of pPPED, pPPEDs, for transfection or bombardment (Figure 1A). The vectors encode, respectively, codon-optimized Cas9(H840A) fusions to the engineered RT and a Gateway destination cassette (Thermo-Fisher) for addition of an RNA module, either pegRNA for PE2 or pegRNA and sgRNA for PE3. We created an entry vector for the RNA modules, pPEG, that allows insertion of a synthetic dsDNA by Golden Gate cloning (Engler et al., 2008) (Figure 1A). pPEG also has a unique restriction enzyme site downstream of the RNA module cloning site and before the *attL2* site for introducing additional elements. To prepare a construct, pegRNA sequence without scaffold (PE2), or pegRNA with scaffold followed by tRNA(Gly) and an sgRNA spacer (PE3), with a *Bsa*I site and compatible sequence at each end, is synthesized and introduced by Golden Gate reaction into pPEG, then the resulting module is transferred by LR recombination into pPPED, pPPEDs, or pPPEM (Figure 1B). Our editing strategy for testing the vectors was PE3. Example peg- and sgRNAs are shown in Figure 1C. A schematic and sequence for preparing RNA modules is given in Figure 1D.

### An Episomal 2-bp Substitution by Agroinfiltation of *N. benthamiana* Leaves

**Figure 2:**
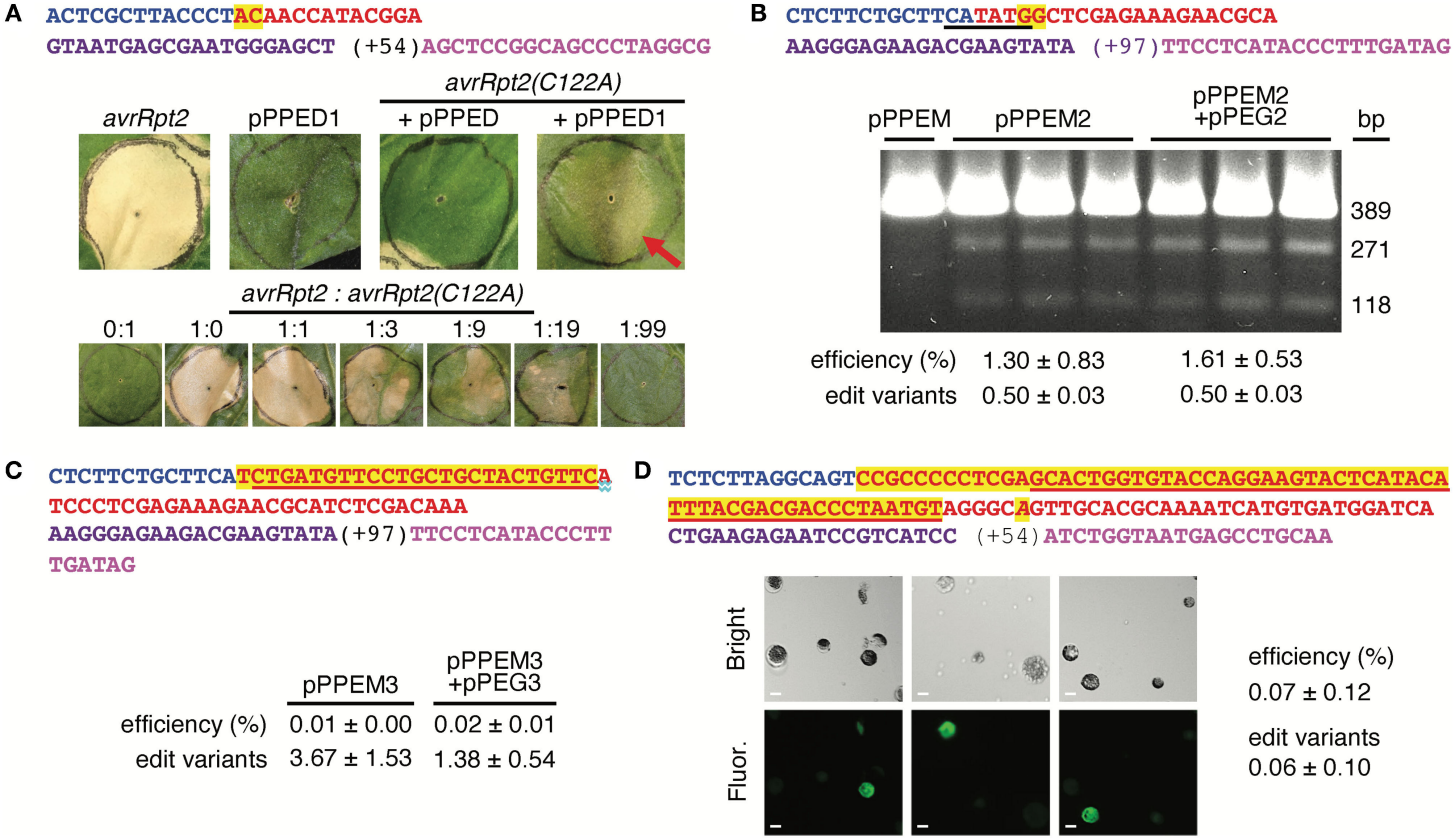
Use of the vectors for spelling changes and tag insertions in three plant species. **(A)** A 2-bp edit of *avrRpt2*(*C122A*) using agroinfiltration of *Nicotiana benthamiana (N. benthamiana)* leaves. Top, features of the pegRNA and sgRNA, colored as in Figure 1C. Middle, leaves 12 days after introduction of *avrRpt2* (OD_600_ = 0.05), or pPPED1, *avrRpt2(C122A*) and empty pPPED, or *avrRpt2(C122A*) and pPPED1 (each at final OD_600_ = 0.5). Bottom, leaves 6 days after infiltration of different ratios of strains delivering *avrRpt2* or *avrRpt2(C122A)*, both at OD_600_ = 0.05 before mixing. These experiments were repeated twice and yielded results consistent with those shown. **(B)** A 2-bp substitution at *OsSULTR3;6* in rice protoplasts. Top, pegRNA and sgRNA features with *Bst*Z17I site underlined. Below, *Bst*Z17I digests of a 389-bp fragment spanning the target amplified from protoplasts transfected with empty pPPEM, pPPEM2, or pPPEM2 and equimolar pPEG2; 271-bp and 118-bp cleavage products indicate the presence of the *Bst*Z17I site introduced by the edit. **(C)** A 25-bp insertion for FLAG tagging at *OsSULTR3;6*. Top, pegRNA and sgRNA features, with FLAG coding sequence underlined. **(D)** A 60-bp insertion for fusion of GFP11 at AT1G26660.1 in Arabidopsis CYTO-sfGFP1-10^OPT^ protoplasts. Top, pegRNA and sgRNA features, with GFP11 coding sequence underlined and a substitution in the pegRNA PAM italicized. Below, bright field and fluorescence micrographs of protoplasts from replicate transfections; scale bar, 10 μm. Bottom of e-g, editing efficiencies determined by amplicon deep-sequencing adjusted for transfection efficiency, and, relative to the number of perfect-edit reads set as 1, the number of edit variant reads (see text). Only perfect-edit reads were counted in determining editing efficiency. Amplicon sequences were analyzed using CRISPResso2 (Clement et al., 2019).

First, we tested pPPED by agroinfiltration of *N. benthamiana* leaves (Figure 2A). The target was a mutated allele of the *avrRpt2* gene of the bacterial plant pathogen *Pseudomonas syringae* (*avrRpt2[C122A]*; (Mazo-Molina et al., 2020), delivered on t-DNA by a co-infiltrated *Agrobacterium* strain. The AvrRpt2 protein elicits programmed cell death in *N. benthamiana*, and the C122A mutation abolishes this activity. The edit, GC to TG at codon 122, would correct the coding sequence to wild type and restore the gene's ability to elicit plant cell death, which can be assessed readily by eye. Together with *avrRpt2(C122A)*, pPPED carrying a pegRNA/sgRNA module for the edit (pPPED1), but not empty pPPED and not pPPED1 alone, resulted in cell death. To estimate efficiency, we determined the sensitivity of the assay by co-infiltrating different ratios of *avrRpt2* and *avrRpt2(C122A)* strains. The *avrRpt2* strain was sufficient for cell death at OD_600_=0.0025 (1:19) but not at OD_600_ = 0.0005 (1:99). Thus, in the editing experiment, in which the *avrRpt2(C122A)* strain was at OD_600_ = 0.5, more than 0.1% (0.0005/0.5) and likely 0.5% (0.0025/0.5) or more of the delivered *avrRpt2(C122A)* was converted to wild type. Amplicon deep sequencing detected only 0.06 ± 0.03% (standard deviation, four infiltrations), likely because the template included *avrRpt2(C122A)* on the vector in *Agrobacterium*, not exposed to the PE reagent.

### Chromosomal 2-bp Substitution and 25-bp Insertion Edits in Rice Protoplasts

Having established the functionality of the dicot binary vector by using agroinfiltration to edit a co-delivered t-DNA, we turned next to the monocot vector, pPPEM, and an endogenous chromosomal target. We tested pPPEM in rice (cv. Nipponbare) protoplasts, targeting two different edits to the bacterial leaf streak disease susceptibility gene *OsSULTR3;6* (LOC_Os01g52130; Cernadas et al., 2014) (Figure 2B). The first edit, GG to CC, eliminates the stop codon and introduces a *Bst*Z17I site. In three transfections with pPPEM carrying the pegRNA/sgRNA module for the edit (pPPEM2), but not in a control transfection with empty pPPEM, *Bst*Z17I digestion of PCR product spanning the target confirmed editing. Amplicon sequencing revealed efficiencies ranging from 0.7 to 2.2%, when adjusted for transfection efficiency (~41%). An equimolar amount of entry vector carrying the RNA module, pPEG2, added to the pPPEM2 transfections did not increase average editing efficiency (unpaired, one tail *t*-test, *p* < 0.05).

The second edit we attempted, at the same location, was a 25-bp insertion for translational fusion of the FLAG epitope (Figure 2C). We carried out three transfections with the editing construct, pPPEM3, and three more with the corresponding pPEG plasmid, pPEG3, added. Amplicon sequencing confirmed insertion, but at relatively low adjusted efficiency, not significantly altered by pPEG3 (0.02 ± 0.01 and 0.01 ± 0.00%, respectively).

### A 66-bp Insertion Allowing Split GFP Tagging in Arabidopsis Protoplasts

Finally, we tested pPPEDs in protoplasts of Arabidopsis lines expressing β strands 1–10 of optimized super-fold green fluorescent protein targeted to the cytoplasm, CYTO-sfGFP1-10^OPT^, or nucleus, NUC-sfGFP1-10^OPT^ (Park et al., 2017). The edit was a 66-bp insertion encoding a linker and GFP11. We reasoned that a split-GFP approach could enable fluorescent tagging despite the apparent insertion size limitation of PE. Indeed, CYTO-sfGFP1-10^OPT^ transfections with a pPPEDs construct, pPPEDs4, targeting the insertion to the cytosolic prefoldin chaperone subunit family protein gene AT1G26660.1 yielded fluorescent protoplasts (Figure 2D), while control transfections of NUC-sfGFP1-10^OPT^ with the same construct, or of CYTO-sfGFP1-10^OPT^ with a pPPEDs construct, pPPEDs5, targeting the insertion to the histone 2B gene (AT5G22880), did not. Transfection of NUC-sfGFP1-10^OPT^ protoplasts with pPPEDs5 targeting the histone 2B gene, though expected to yield fluorescence, did not detectably do so. The pPPEDs4 RT template includes a C to A substitution 6 bp after the GFP11 sequence that destroys the pegRNA PAM, a strategy proposed to limit indel formation between the PE3 nicks and to disfavor reversion of the edited strand (Anzalone et al., 2019). Amplicon sequencing of the CYTO- and NUC-sfGFP1-10^OPT^ transfections with pPPEDs4 (three each) confirmed successful insertion, averaging 0.07 ± 0.12% adjusted efficiency.

### Editing Efficiencies

For all edits, the positive amplicon reads included some with other differences from the original sequence in the window encompassing the nick sites and edit plus 2 bp on either side, and some of the insertion edit reads had one or more substitutions or indels in the insertion. The frequencies of these “edit variants” (combined) are given in Figure 2. Edit variants were not counted in the reported efficiencies. They may represent non-templated changes during DNA repair, spontaneous mutations, or PCR or sequencing artifact. Notably, in the 66-bp insertion experiment in Arabidopsis protoplasts, sequencing of the AT1G26660.1 amplicon from negative control transfection of CYTO-sfGFP1-10^OPT^ with pPPEDs5, and from a transfection of NUC-GFP1-10^OPT^ with pPPEDs5, yielded an average of 6.4 ± 4.0% reads varying from the original sequence. This relatively high background suggests that editing efficiencies in this and the other experiments may have been higher than we calculated counting only perfect reads. Sequence variants without the edit but with an indel or substitution appearing to have arisen due to imperfect non-homologous end joining of a double strand break, presumably resulting from the pegRNA- and sgRNA-mediated nicks together, were detected in all of the sequenced test samples, at high frequencies relative to the edit (Supplementary Figure 1).

For the 2-bp substitution edits, it is possible that some of the perfect-edit reads resulted from substitutions that exactly duplicate the intended edit but that occurred by chance during repair of the cut DNA, or during PCR amplification or sequencing. To examine this possibility, we searched the variant reads from the 25-bp edit at *OsSULTR3;6* for any that by chance match the perfect-edit sequence for the 2-bp substitution at *OsSULTR3;6*, which was targeted to precisely the same nick site. Across the six total pPEM3 and pPEM3 plus pPEG3 sequence sets, an average of 0.04 ± 0.01% of the reads matched the perfect-read sequence for the 2-bp edit (Supplementary Table 3). This frequency is 17- to 55-fold lower than the frequencies of perfect-edit reads in the amplicon sequences from the actual 2-bp edit experiments (pPEM2 and pPEM2 plus pPEG2, above). Thus, contribution of non-templated substitutions, or PCR or sequencing artifact to the calculated efficiencies for the smaller edits can be considered negligible.

For the 25-bp and 66-bp insertion edits, the observation of fluorescent protoplasts for the latter notwithstanding, it is conceivable that the small numbers of positive reads are artifact resulting from template switching during PCR amplification. Template switching, first described in the 1990's (Paabo et al., 1990; Odelberg et al., 1995) has been found to be a rare source of erroneous, chimeric reads in high throughput sequence sets (Kebschull and Zador, 2015). In each of the two insertion-edit amplicon sequence sets, since the primers used anneal to the genomic DNA and not to the construct, two template switches would have had to occur for artifactual positive reads to have been generated, which can be expected to be exceedingly rare. Nonetheless, to control for the possibility in each case, we deep-sequenced amplicon generated from a mixed template of untransfected protoplast DNA and a 2-fold higher molar amount of the editing construct, 40 ng rice cv. Nipponbare DNA with pPPEM3 for the 25-bp edit and 10 ng Arabidopsis CYTO-sfGFP1-10^OPT^ DNA with pPPEDs4 for the 66-bp edit. None of the resulting aligned reads (averaging 16,672 and 46,631 reads, respectively, across two replicates each) contained the respective insertion sequences, perfect or variant.

## Summary

In summary, we developed vectors for straightforward plant PE construct assembly and demonstrated their efficacy in one monocot and two dicot species. Edits included two 2-bp codon changes, a 25-bp FLAG tag insertion, and a 66-bp GFP11 insertion. The 66-bp insertion is the largest reported for PE and provides important proof of concept for fluorescent tagging using PE. Editing efficiencies, especially for insertions, were low. However, efficiencies are likely to be higher in stably transformed plants or with meristem transformation (Maher et al., 2020), and possibly with optimization of RT template and PBS length (Lin et al., 2020; Tang et al., 2020; Xu et al., 2020a), and the vectors thus useful in extending PE to diverse plant species.

## Data Availability Statement

The datasets presented in this study can be found in online repositories. The names of the repository/repositories and accession number(s) can be found below: < https://www.ncbi.nlm.nih.gov/, PRJNA641949>.

## Author Contributions

LW, HK, NZ, RR, MW, AR, FM, JL, GM, and AB conceived and designed the study. LW, NZ, RR, and MW performed the experiments. LW, HK, NZ, RR, MW, SC, ZF, GM, and AB analyzed data. All authors contributed to preparation of the manuscript.

## Conflict of Interest

The authors declare that the research was conducted in the absence of any commercial or financial relationships that could be construed as a potential conflict of interest.
