# Supplementary material for: Spelling Changes and Fluorescent Tagging With Prime Editing Vectors for Plants
Source: Front Genome Ed. 2021 Mar 4;3:617553. doi: 10.3389/fgeed.2021.617553 (PMC8525380; doi:10.3389/fgeed.2021.617553)
Supplement: Supplementary file 1 [file Data_Sheet_1.PDF]

# *Supplementary Material*

## 1 Supplementary Figures

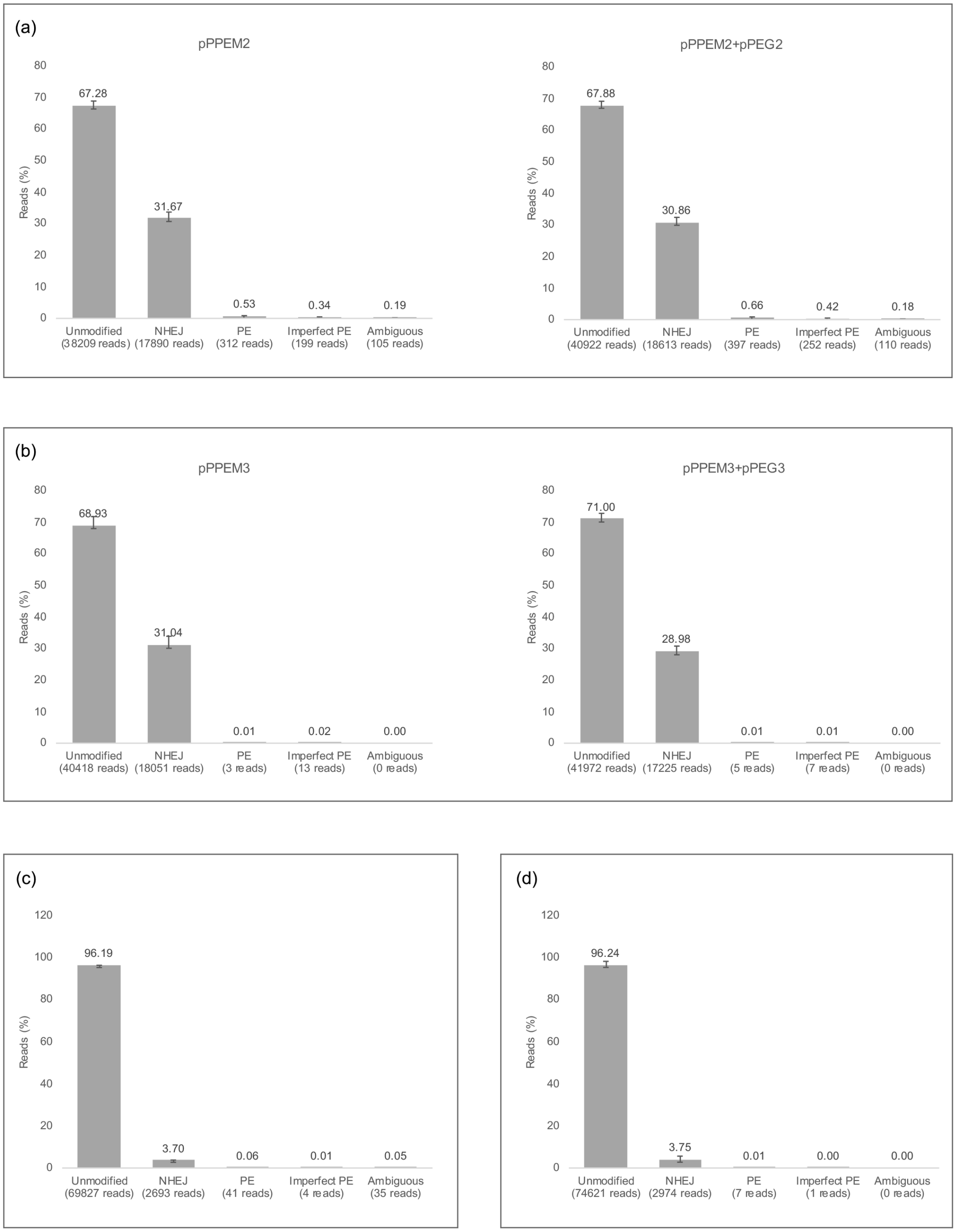

**Supplementary Figure 1.** Amplicon sequence read frequencies by read type. Panels show frequencies of reads classified using CRISPResso2 for (a) the 2-bp substitution at *OsSULTR3;6* in rice, (b) the 25-bp insertion for FLAG tagging at *OsSULTR3;6* in rice, (c) the 2-bp substitution at *avrRpt2*(*C122A*) in *Nicotiana benthamiana* and (d) the 66-bp insertion for fusion of GFP11 at AT1G26660.1 in Arabidopsis. UNMODIFIED, no difference from the original sequence; NHEJ, indel or substitution but not the intended edit; PE, the intended edit and no other difference; Imperfect PE, the intended edit with at least one other difference (indel or substitution); Ambiguous, not clearly close to the original or edited sequences.

## 2 Supplementary Tables

**Supplementary Table 1.** Primers used for PCR amplification of PE targets.

| Name | Sequence (5′ to 3′) | Purpose[a] |
|---|---|---|
| AvrRpt2_adaptor_F | TCGTCGGCAGCGTCAGATGTGTATAAGAGACAG CGCATTACTCGCTACTAAGACAG | *avrRpt2(C122A)* amplicon sequencing |
| AvrRpt2_adaptor_R | GTCTCGTGGGCTCGGAGATGTGTATAAGAGACA GGGTCTACCCGAGTTAATCCTTC | *avrRpt2(C122A)* amplicon sequencing |
| AT1G26660.1_GENEWIZadaptor_F | GACTGGAGTTCAGACGTGTGCTCTTCCGATCTC CAACAGTTTGGTCCATTCCG | *AT1G26660.1* amplicon sequencing |
| AT1G26660.1_GENEWIZadaptor_R | ACACTCTTTCCCTACACGACGCTCTTCCGATCT TGCGTAGGAACCCTGCATC | *AT1G26660.1* amplicon sequencing |
| sultr3;6_F | GTCTGGATACGTCAGGGACAT | *OsSULTR3;6* amplicon digests |
| sultr3;6_R | ATTCCCCATTGCCCCTAATCA | *OsSULTR3;6* amplicon digests |
| sultr3;6_adaptor_F | TCGTCGGCAGCGTCAGATGTGTATAAGAGACAG GTCTGGATACGTCAGGGACAT | *OsSULTR3;6* amplicon sequencing |
| sultr3;6_adaptor_R | GTCTCGTGGGCTCGGAGATGTGTATAAGAGACA GATTCCCCATTGCCCCTAATCA | *OsSULTR3;6* amplicon sequencing |
| sultr3;6_GENEWIZadaptor_F | ACACTCTTTCCCTACACGACGCTCTTCCGATCT GTCTGGATACGTCAGGGACAT | *OsSULTR3;6* amplicon sequencing [a] |
| sultr3;6_GENEWIZadaptor_R | GACTGGAGTTCAGACGTGTGCTCTTCCGATCT ATTCCCCATTGCCCCTAATCA | *OsSULTR3;6* amplicon sequencing [a] |

[a] These duplicate sultr3;6 amplicon sequencing primers were made with the GENEWIZ adaptors (at the 5 ′ ends) and were used for the template switching control experiment, which was performed after the initial editing experiments. Using the GENEWIZ adaptors reduced cost.

**Supplementary Table 2.** Transfection efficiency, percent perfect-edit reads determined by amplicon deep-sequencing, and calculated editing efficiency, averaged across replicates.

| Edit | Construct(s) | Transfection Efficiency (%) | Perfect Reads (%)[a] | Calculated Editing Efficiency (%)[b] |
|---|---|---|---|---|
| 2-bp substitution at *avrRpt2(C122A)* | pPPED1 | Not applicable | 0.057 | 0.057 |
| 2-bp substitution at *OsSULTR3;6* | pPPEM2 | 0.408 | 0.529 | 1.297 |
| | pPPEM2+pPEG2 | 0.408 | 0.659 | 1.615 |
| | pPPEM3 | 0.408 | 0.006 | 0.014 |

| | | | | |
|---|---|---|---|---|
| 25-bp FLAG tag insertion at *OsSULTR3;6* | pPPEM3+pPEG3 | 0.408 | 0.008 | 0.019 |
| 66-bp GFP11 insertion at *AT1G26660.1* | pPPEDs4 | 0.116 | 0.008 | 0.066 |

[a] Percent of the total number of mapped reads.

[b] Percent perfect-edit reads divided by transfection efficiency.

**Supplementary Table 3.** Reads from the 25-bp editing experiments matching the perfect-edit read sequence for the 2-bp edit.

| | **pPEM3 #1** | **pPEM3 #2** | **pPEM3 #3** | **pPEM3 and pPEG3 #1** | **pPEM3 and pPEG3 #3** | **pPEM3 and pPEG3 #3** |
|---|---|---|---|---|---|---|
| **Total mapped reads** | 53,854 | 67,421 | 54,179 | 65,941 | 55,841 | 55,844 |
| **Matching reads** | 25 | 29 | 24 | 33 | 18 | 8 |
| **% of total** | 0.046 | 0.043 | 0.044 | 0.050 | 0.032 | 0.014 |
